# Supplementary material for: Direct gas-phase formation of formic acid through reaction of Criegee intermediates with formaldehyde
Source: Commun Chem. 2023 Jun 22;6:130. doi: 10.1038/s42004-023-00933-2 (PMC10287650; doi:10.1038/s42004-023-00933-2)
Supplement: Supplementary file 2 — Supplementary Information [file 42004_2023_933_MOESM2_ESM.pdf]

Supplementary Information for "Direct gas-phase formation of formic acid through reaction of Criegee intermediates with formaldehyde"

Pei-Ling Luo[1]*, I-Yun Chen[1], M. Anwar H. Khan[2] & Dudley E. Shallcross[2]

[1]Institute of Atomic and Molecular Sciences, Academia Sinica, Taipei 106319, Taiwan.
[2]School of Chemistry, Cantock's Close, University of Bristol, Bristol BS8 1TS, U.K.
*corresponding author: plluo@gate.sinica.edu.tw

Table of content

Supplementary Note 1. Theoretical investigations of the CH_2OO + HCHO reaction.
Supplementary Figure 1. Enthalpy profiles of the reaction between CH_2OO and HCHO.
Supplementary Figure 2. Schematic of the experimental setup.
Supplementary Figure 3. Obtained time traces of CH_2OO (open circles) and kinetic model fitted curves (solid lines).
Supplementary Figure 4. Comparison of plots of k_{obs} vs. $[HCHO]_0$ derived by kinetic model fit and single-exponential fit.
Supplementary Figure 5. Difference absorbance spectra of HCOOH.
Supplementary Figure 6. Difference absorbance spectra of CO.
Supplementary Figure 7. Fractional contribution of each species to the total loss rate of CH_2OO by reaction with HCHO, H_2O, and $(H_2O)_2$, as a function of relative humidity and temperature.
Supplementary Figure 8. The concentration of HCOOH at different altitudes obtained by the Canadian ACE satellites.
Supplementary Table 1. Summary of experimental conditions and obtained rate coefficients k_{obs}.
Supplementary Table 2. Summary of experimental and theoretical results of the rate coefficient for the reactions of CH_2OO with HCHO, CH_3CHO, and CH_3COCH_3.
Supplementary Table 3. Kinetic model and rate coefficients employed for product analysis.
Supplementary Table 4. Summary of experimental conditions, fitting parameters, and branching yields of HCOOH and CO.
Supplementary References

Supplementary Note 1. Theoretical investigations of the CH_2OO + HCHO reaction.

The transition states and intermediates of the reaction CH_2OO + HCHO have been studied based on various quantum-chemical calculations.[1−3] Long *et al.* investigated the reaction kinetics between CH_2OO and other atmospheric species including HCHO by applying the W3X-L//CCSD(T)-F12a/cc-pVDZ-F12 method and indicated that the rate coefficient of the reaction CH_2OO + HCHO has a strong negative temperature dependence in the range 250−350 K.[1] Jalan *et al.* employed the CCSD(T)/B3LYP to calculate the zero-point corrected relative energies of the transition states and intermediates corresponding to the reactants. They also calculated the temperature- and pressure-dependent rate coefficients and product yields of the reaction CH_2OO + HCHO by applying the master equation model and the reservoir state method.[2] Elakiya *et al.* performed the calculation on the kinetics and products for the reaction between CH_2OO and HCHO with the M06-2X/6-311++G(d,p) method.[3] We intergrated results of these three reports to present the enthalpy profiles of the reaction CH_2OO + HCHO, as shown in Supplementary Fig. 1. The energies of C1, TS1 and SOZ (secondary ozonide) were predicted to be −6.9 (−6.30), −6.3 (−5.31) and −51.3 (−49.54) kcal mol^{-1}, respectively, relative to CH_2OO + HCHO; here the values are taken from Jalan *et al.*[2] and those in parentheses are from Long *et al.*[1] A discrepency of ~1 kcal mol^{-1} for the relative energy of TS1 between the two theoretical predictions is observed. In addition, according to the potential energy surface calculated by Jalan *et al.*, SOZ can only be stablized at very high pressure (> a few atm). At low pressure condition (< 1 atm), SOZ will first form hydroxymethylformate (HMF) (−128.2 kcal mol^{-1}) via TS3 (−22.6 kcal mol^{-1}) and then undergo the H-migration via TS4 (−107.4 kcal mol^{-1}) to produce *trans*-HCOOH and HCHO or it may directly decompose to generate the high-energy *cis*-HCOOH + HCHO via TS2 (−9.6 kcal mol^{-1}).[2] In contrast to Jalan *et al.*, Elakiya *et al.* predicted that SOZ can decompose to form HCOOH + HCHO (−67.83 kcal mol^{-1}) and CO + H_2O + HCHO (−63.81 kcal mol^{-1}) via TS5 (56.91 kcal mol^{-1}) and TS6 (3.07 kcal mol^{-1}), respectively.[3] Because of the high energy barrier between the SOZ and the TS5, Elakiya *et al.* concluded that the formation of products HCOOH + HCHO is unfavourable and the dominant product channel is the formation of CO + H_2O + HCHO.[3]

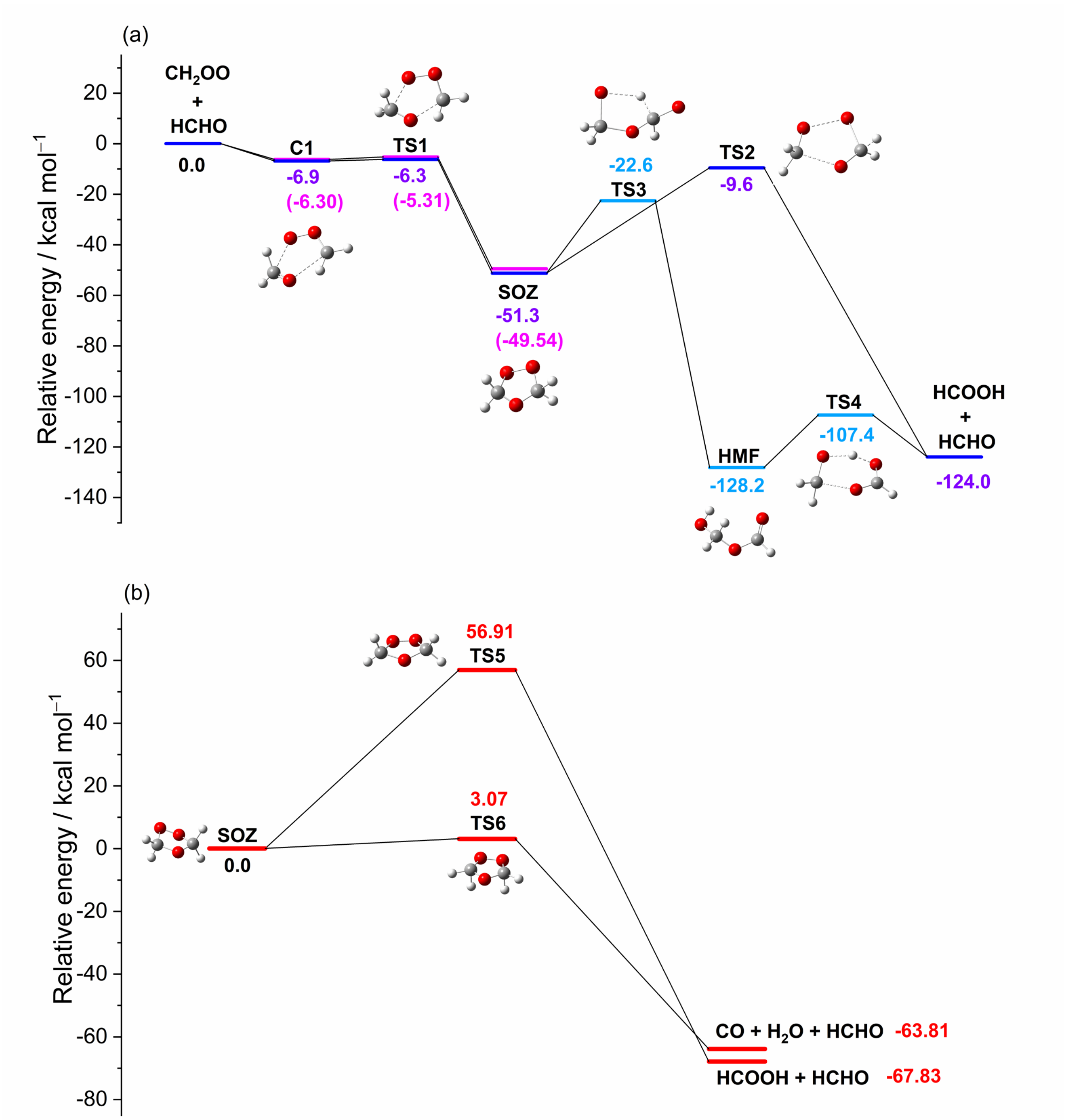

Supplementary Figure 1. Enthalpy profiles (ΔH) of the reaction between CH_2OO and HCHO. Energies and the notations of the intermediates are taken from the work of Long *et al.*[1] (pink), Jalan *et al.*[2] (purple and blue) and Elakiya *et al.*[3] (red). (a) The values in parentheses from Long *et al.* (pink) were calculated by the W3X-L//CCSD(T)-F12a/cc-pVDZ-F12 method at 0 K, relative to the reactants. The values from Jalan *et al.* (purple and blue) were calculated relative to the reactants, by CCSD(T)/B3LYP. (b) Enthalpies from Elakiya *et al.* (red) were calculated by the M06-2X/6-311++G(d,p) method at 298 K, relative to the SOZ.

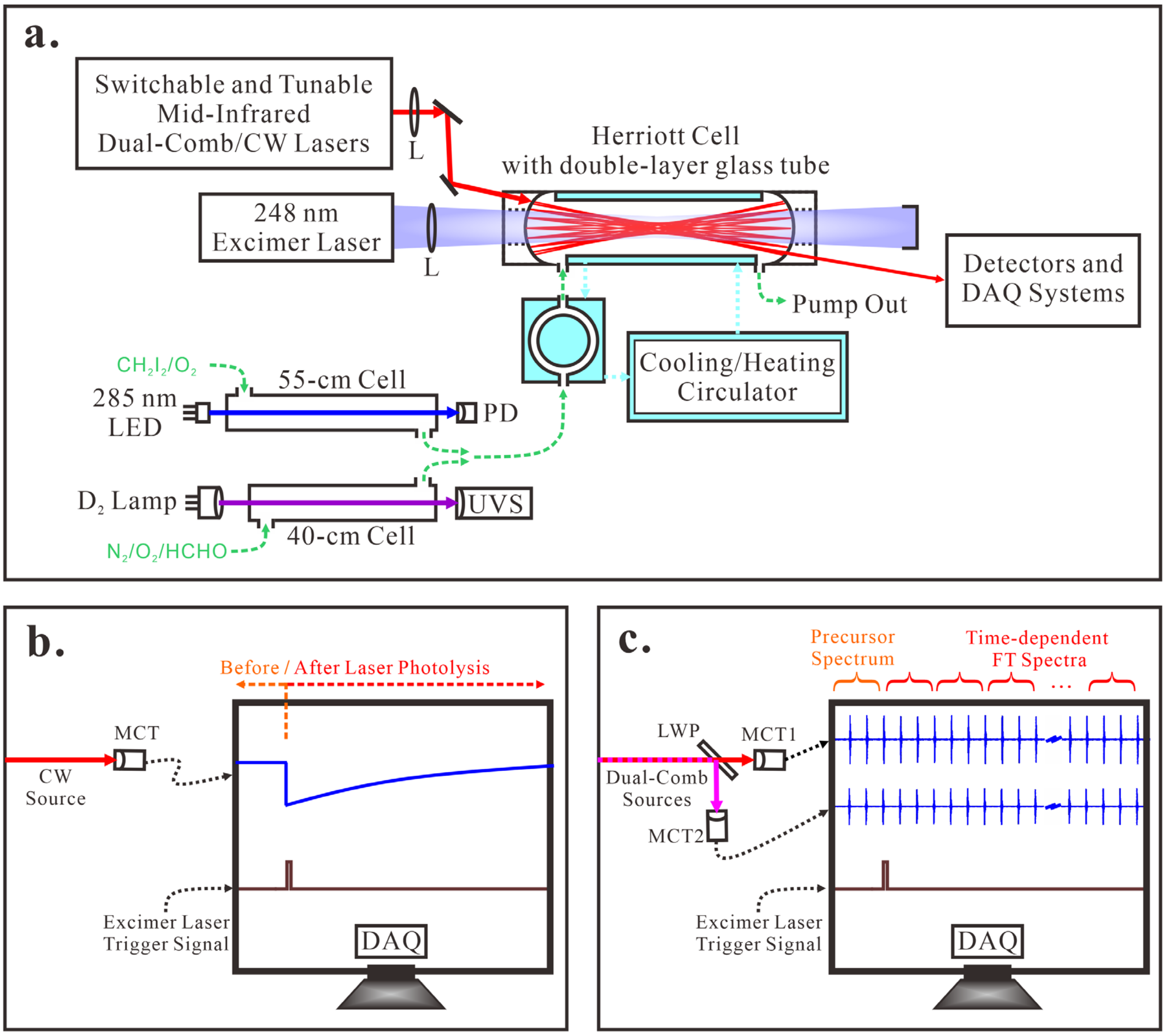

Supplementary Figure 2. Schematic of the experimental setup. (a) An overview of the entire experimental system for reaction kinetics and product studies. The mid-infrared dual-comb spectrometers are designed with switchable dual-comb and continuous-wave (cw) operation modes. A Herriott cell is constructed by using a pair of 2-inch concave gold mirrors with a 25-mm-diameter center hole and a 4-mm-diameter off-axis hole. A double-layer glass tube as the main body of the cell can be temperature controlled by a liquid circulator. The Herriott cell is designed to allow 63 passes of the infrared beam. The center hole is used for passage of the 248-nm photolysis beam. The distance between the two mirrors is 655 mm and the total path length of infrared beam is estimated to be 41.8 m. The overlapping path between infrared and photolysis beams is estimated to be 13.4 m. (b) Schematic diagram of the experiments with cw source for kinetic measurements. (c) Schematic diagram of the experiments with dual-comb sources for determination of branching yields of reaction products. Here, DAQ: data-acquisition board, LED: light-emitting diode, PD: photodiode, UVS: ultraviolet spectrometer, LWP: longwave pass filter, and MCT: HgCdTe detector.

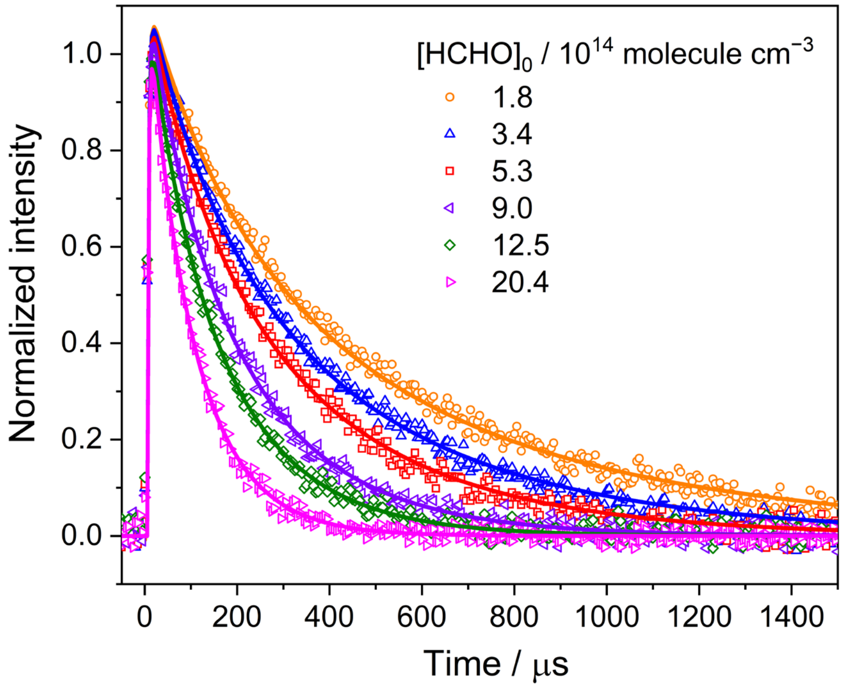

Supplementary Figure 3. Obtained time traces of CH_2OO (open circles) and kinetic model fitted curves (solid lines). The data correspond to experiment 1–6 listed in Supplementary Table 1. The derived first-order rate coefficients as a function of $[HCHO]_0$ are shown in Supplementary Fig. 4 and are compared to the value obtained by using single exponential fit.

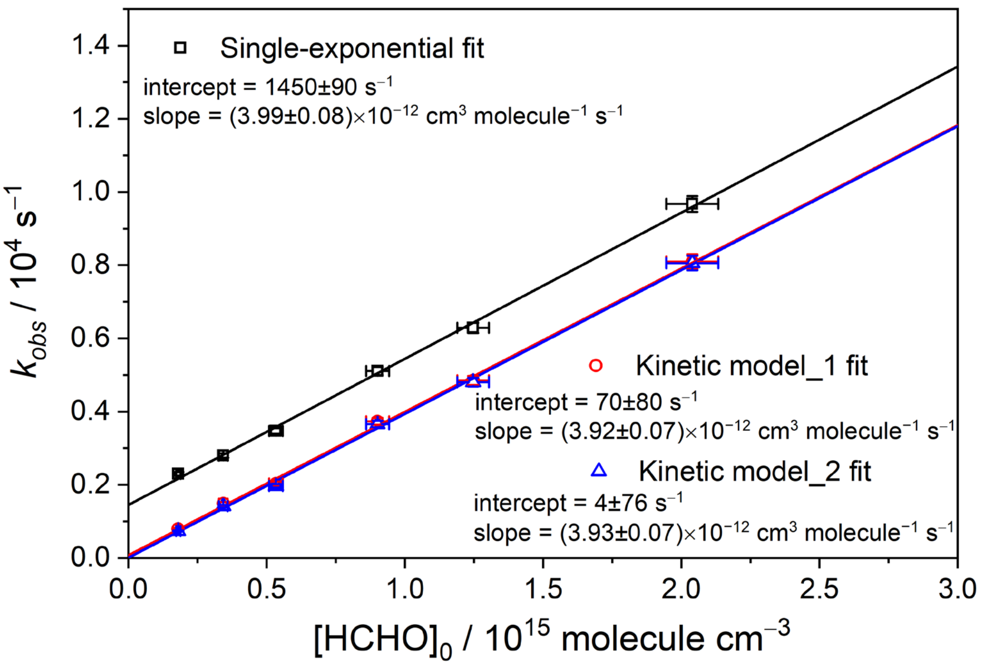

Supplementary Figure 4. Comparison of plots of k_{obs} vs. $[HCHO]_0$ derived by kinetic model fit and single-exponential fit. The kinetic model_1 takes into account key reaction paths including the formation and self-reaction of CH_2OO as well as the CH_2OO + I reaction, but without taking into account the reaction of CH_2OO + HCOOH. The kinetic model_2 takes into account the effect from the reaction of CH_2OO + HCOOH and the yield of HCOOH formed from the reaction CH_2OO + HCHO is assumed to be 50%. The fitted slopes corresponding to the second-order rate constants $k_{CH2OO+HCHO}$ are consistent within the errors, indicating the reaction kinetics were measured under pseudo-first order conditions. The intercept obtained by fitting the data derived using single-exponential fit represents the loss rate of CH_2OO by the CH_2OO self-reaction and reactions of CH_2OO with other species generated in the reaction system.

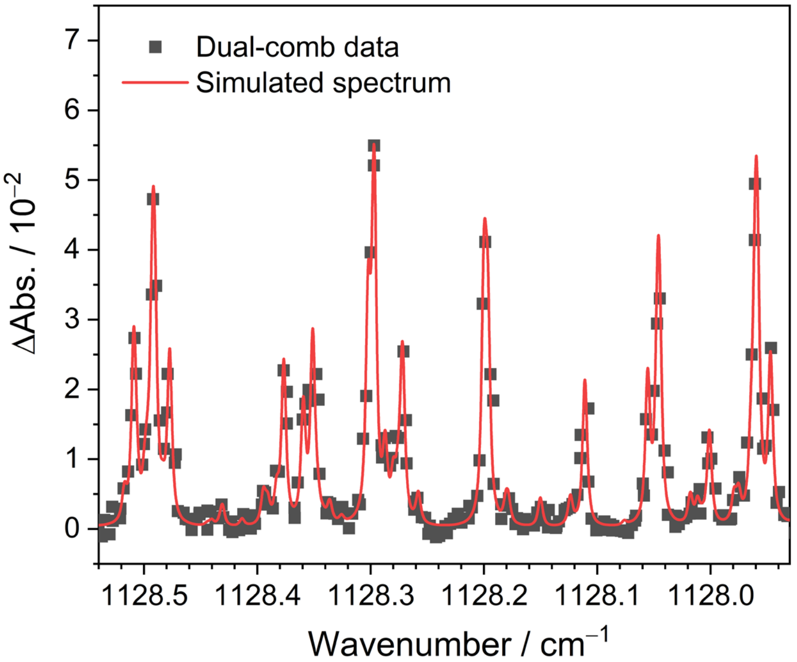

Supplementary Figure 5. Difference absorbance spectra of HCOOH. The dual-comb spectra were measured using the dual-comb spectrometer with different spectral sampling spacings of 181, 210, and 288 MHz at 12.48–12.54 ms after laser photolysis of a flowing mixture of $CH_2I_2/HCHO/O_2/N_2$ (0.016/0.123/14.9/0.04 Torr, P_T = 15.1 Torr, 296 K). The simulated spectrum of HCOOH is taken from the HITRAN database[4] with the parameters: L_{eff} = 1340 cm, P_T = 15.1 Torr, T = 296 K, and [HCOOH] = 1.06×10^{13} molecule cm^{-3}. Here, the data correspond to the experiment 1 listed in Supplementary Table 3.

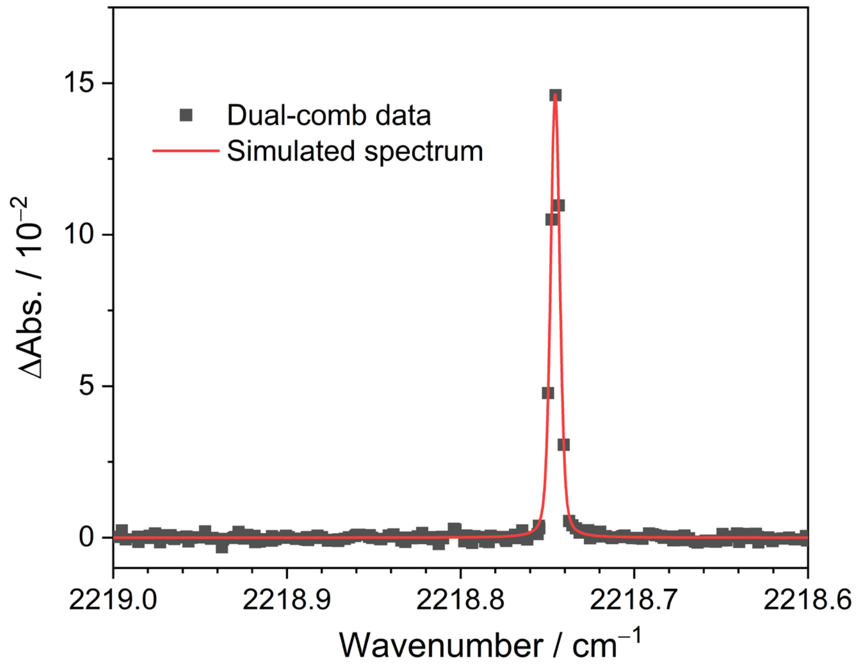

Supplementary Figure 6. Difference absorbance spectra of CO. The dual-comb spectra were measured using the dual-comb spectrometer with different spectral sampling spacings of 181, 210, and 288 MHz at 12.48–12.54 ms after laser photolysis of a flowing mixture of $CH_2I_2/HCHO/O_2/N_2$ (0.016/0.123/14.9/0.04 Torr, P_T = 15.1 Torr, 296 K). The simulated spectrum of CO is taken from the HITRAN database[4] with the parameters: L_{eff} = 1340 cm, P_T = 15.1 Torr, T = 296 K, and [CO] = 2.90×10^{13} molecule cm^{-3}. Here, the data correspond to the experiment 1 listed in Supplementary Table 3.

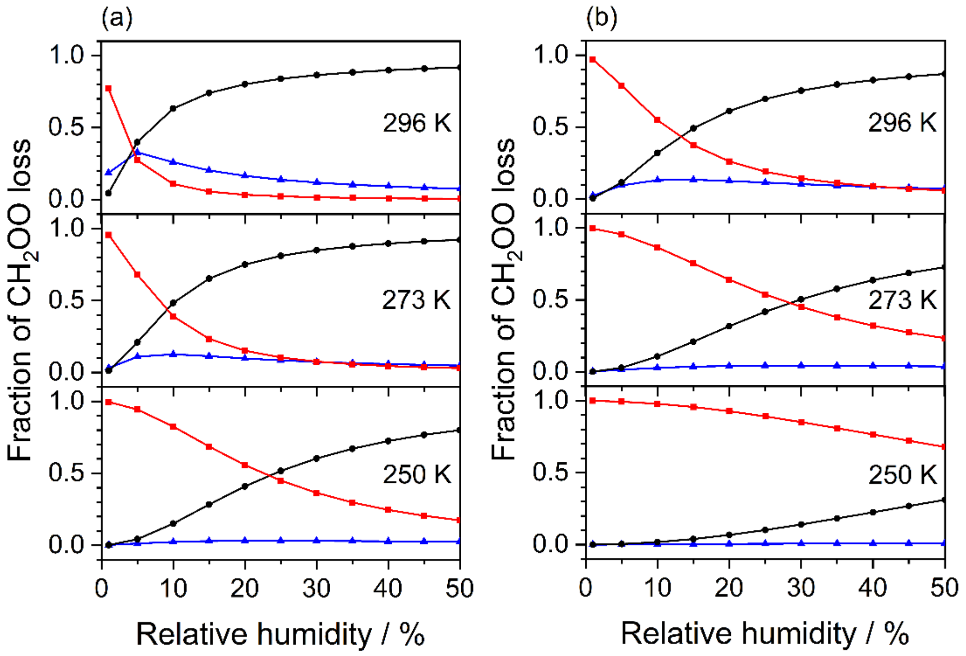

Supplementary Figure 7. Fractional contribution of each species to the total loss rate of CH_2OO by reaction with HCHO (red), H_2O (blue), and $(H_2O)_2$ (black), as a function of relative humidity and temperature. Here, the concentrations of HCHO are set to be (a) 0.1 ppm and (b) 1 ppm at 1 atm. Here, the concentrations of H_2O are obtained by using the relative humidity measured by Schuyler *et al.*[5] and the Arden Buck equation: $P_s(T')=6.1121\times\exp((18.678-T'/234.5)(T'/(257.14+T')))$, where $P_s(T')$ is the saturation water vapor pressure in hPa and T' is the air temperature in °C.[6] The concentrations of water dimer are calculated by applying the equilibrium constant for $2\ (H_2O)_{(g)} \rightarrow (H_2O)_{2(g)}$.[7] The bimolecular rate constants of $CH_2OO + H_2O$ and $CH_2OO + (H_2O)_2$ are calculated from the results by Lin *et al.*[8]

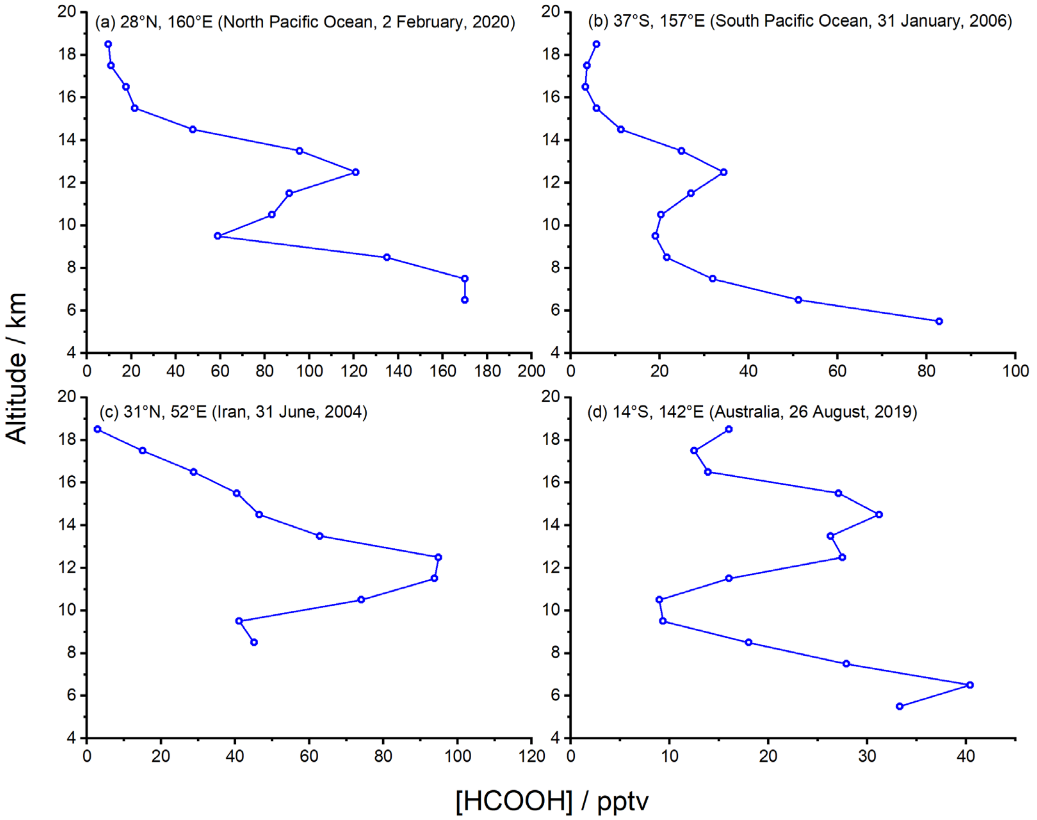

Supplementary Figure 8. The concentration of HCOOH at different altitudes taken from the ACE-FTS data that measured by the Canadian ACE satellites[9].

Supplementary Table 1. Summary of experimental conditions and obtained rate coefficients k_{obs}.

Set	Expt.	Temp. / K	P_T / Torr	$[CH_2OO]_0$ [b] / 10^{12} [a]	$[HCHO]_0$ [c] / 10^{14} [a]	k_{obs} [d] / 10^3 s^{-1}
1	1	296	13.3	7.4	9.0	8.44
	2	296	13.3	7.4	5.3	7.14
	3	296	13.3	7.4	3.4	5.77
	4	296	13.3	7.4	1.8	4.17
	5	296	13.3	7.4	20.4	11.06
	6	296	13.3	7.4	12.5	7.70
	7	296	13.3	7.4	17.6	5.11
	8	296	13.3	7.4	14.3	3.47
	9	296	13.3	7.4	10.8	2.80
	10	296	13.3	7.4	7.3	2.30
	11	296	13.3	7.4	23.7	9.67
	12	296	13.3	7.4	16.0	6.29
2	13	296	6.4	8.6	7.2	3.87
	14	296	6.4	8.6	9.6	4.91
	15	296	6.4	8.6	11.6	5.61
	16	296	6.4	8.6	14.4	6.92
	17	296	6.4	8.6	18.7	8.45
	18	296	6.4	8.6	22.7	9.97
	19	296	6.4	8.6	27.1	11.95
	20	296	6.4	8.6	30.9	13.50
	21	296	6.4	8.6	22.3	9.96
	22	296	6.4	8.6	18.3	8.47
	23	296	6.4	8.6	13.7	6.43
	24	296	6.4	8.6	9.4	4.84
	25	296	6.4	8.6	4.7	2.92
	26	296	6.4	8.6	2.5	2.15
	27	296	6.4	8.6	27.1	11.90
	28	296	6.4	8.6	30.7	13.42
	29	296	6.4	8.6	34.5	14.86
3	30	296	56.0	7.0	9.4	5.85
	31	296	56.0	7.0	10.8	6.22
	32	296	56.0	7.0	13.1	7.25
	33	296	56.0	7.0	8.3	5.28
	34	296	56.0	7.0	7.0	4.90
	35	296	56.0	7.0	5.6	4.10

	36	296	56.0	7.0	4.1	3.36
4	37	296	46.0	5.5	13.9	6.85
	38	296	46.0	5.5	11.4	5.38
	39	296	46.0	5.5	8.7	4.52
	40	296	46.0	5.5	5.8	3.39
	41	296	46.0	5.5	4.4	2.92
	42	296	46.0	5.5	2.9	2.18
5	43	296	33.0	6.3	17.2	8.48
	44	296	33.0	6.3	14.7	7.47
	45	296	33.0	6.3	11.9	6.33
	46	296	33.0	6.3	9.1	5.05
	47	296	33.0	6.3	6.1	3.84
	48	296	33.0	6.3	2.8	2.48
6	49	296	20.0	6.8	21.6	10.48
	50	296	20.0	6.8	18.3	8.87
	51	296	20.0	6.8	16.0	7.73
	52	296	20.0	6.8	12.9	6.47
	53	296	20.0	6.8	9.9	5.41
	54	296	20.0	6.8	6.6	4.06
	55	296	20.0	6.8	3.2	2.76
7	56	296	21.4	6.0	33.7	15.36
	57	296	21.4	6.0	28.2	12.44
	58	296	21.4	6.0	19.5	9.46
	59	296	21.4	6.0	13.3	6.57
	60	296	21.4	6.0	6.9	4.32
	61	296	21.4	6.0	10.1	5.46
	62	296	21.4	6.0	16.5	8.41
	63	296	21.4	6.0	24.0	11.27
8	64	288.3	21.4	6.3	34.3	16.21
	65	288.3	21.4	6.3	28.6	13.64
	66	288.3	21.4	6.3	19.8	9.42
	67	288.3	21.4	6.3	13.7	7.19
	68	288.3	21.4	6.3	7.0	4.19
	69	288.3	21.4	6.3	10.8	5.80
	70	288.3	21.4	6.3	17.6	8.58
	71	288.3	21.4	6.3	24.3	11.57
9	72	307.7	21.4	5.7	32.0	13.19
	73	307.7	21.4	5.7	26.8	11.59

	74	307.7	21.4	5.7	18.7	7.96
	75	307.7	21.4	5.7	12.6	6.19
	76	307.7	21.4	5.7	6.5	3.59
	77	307.7	21.4	5.7	9.5	5.35
	78	307.7	21.4	5.7	15.7	7.01
	79	307.7	21.4	5.7	22.8	9.46
10	80	317.5	21.4	6.5	31.0	12.25
	81	317.5	21.4	6.5	26.3	10.85
	82	317.5	21.4	6.5	18.1	8.09
	83	317.5	21.4	6.5	12.5	6.08
	84	317.5	21.4	6.5	6.3	3.82
	85	317.5	21.4	6.5	9.4	4.84
	86	317.5	21.4	6.5	15.3	6.72
	87	317.5	21.4	6.5	22.4	9.14
11	88	327	21.4	5.3	30.0	11.04
	89	327	21.4	5.3	25.3	9.31
	90	327	21.4	5.3	17.4	6.73
	91	327	21.4	5.3	11.9	5.60
	92	327	21.4	5.3	5.9	3.36
	93	327	21.4	5.3	8.8	4.34
	94	327	21.4	5.3	14.6	6.06
	95	327	21.4	5.3	21.4	8.35
12	96	336.5	21.4	5.1	29.3	9.66
	97	336.5	21.4	5.1	24.5	8.51
	98	336.5	21.4	5.1	17.1	5.86
	99	336.5	21.4	5.1	11.8	4.87
	100	336.5	21.4	5.1	6.0	2.91
	101	336.5	21.4	5.1	8.9	3.91
	102	336.5	21.4	5.1	14.4	5.46
	103	336.5	21.4	5.1	21.0	7.18
13	104	277.5	14.1	6.1	37.9	20.33
	105	277.5	14.1	6.1	31.6	17.06
	106	277.5	14.1	6.1	21.8	12.33
	107	277.5	14.1	6.1	14.8	8.03
	108	277.5	14.1	6.1	7.5	4.62
	109	277.5	14.1	6.1	10.5	6.49
	110	277.5	14.1	6.1	17.9	9.74
	111	277.5	14.1	6.1	26.7	14.66

14	112	277.5	21.3	6.4	34.7	19.08
	113	277.5	21.3	6.4	29.4	16.29
	114	277.5	21.3	6.4	20.0	10.95
	115	277.5	21.3	6.4	13.4	7.65
	116	277.5	21.3	6.4	6.7	4.40
	117	277.5	21.3	6.4	9.8	5.83
	118	277.5	21.3	6.4	16.6	9.83
	119	277.5	21.3	6.4	24.6	13.83
15	120	283	21.3	6.3	34.4	17.70
	121	283	21.3	6.3	28.7	15.58
	122	283	21.3	6.3	19.7	10.67
	123	283	21.3	6.3	13.5	7.34
	124	283	21.3	6.3	6.9	4.25
	125	283	21.3	6.3	9.7	5.84
	126	283	21.3	6.3	16.5	9.04
	127	283	21.3	6.3	24.3	12.48
16	128	283	14.0	5.8	36.6	18.18
	129	283	14.0	5.8	30.5	15.53
	130	283	14.0	5.8	21.1	10.45
	131	283	14.0	5.8	14.1	7.22
	132	283	14.0	5.8	6.9	4.31
	133	283	14.0	5.8	10.0	5.55
	134	283	14.0	5.8	17.2	8.73
	135	283	14.0	5.8	25.6	12.95
17	136	288	14.0	5.8	37.6	17.67
	137	288	14.0	5.8	30.3	14.58
	138	288	14.0	5.8	20.7	9.68
	139	288	14.0	5.8	14.2	7.03
	140	288	14.0	5.8	7.1	4.30
	141	288	14.0	5.8	10.1	5.36
	142	288	14.0	5.8	17.1	8.19
	143	288	14.0	5.8	25.7	11.89
18	144	288	21.3	6.1	33.3	16.64
	145	288	21.3	6.1	27.8	13.61
	146	288	21.3	6.1	19.0	9.47
	147	288	21.3	6.1	12.8	6.94
	148	288	21.3	6.1	6.2	4.55
	149	288	21.3	6.1	9.1	5.10

	150	288	21.3	6.1	15.2	7.71
	151	288	21.3	6.1	23.2	11.93
19	152	318	14.0	4.0	32.7	11.92
	153	318	14.0	4.0	27.4	10.24
	154	318	14.0	4.0	18.7	6.86
	155	318	14.0	4.0	12.7	5.29
	156	318	14.0	4.0	6.3	3.10
	157	318	14.0	4.0	9.3	4.08
	158	318	14.0	4.0	15.2	6.53
	159	318	14.0	4.0	23.0	9.03
20	160	336.5	14.0	3.1	30.0	9.35
	161	336.5	14.0	3.1	25.1	7.78
	162	336.5	14.0	3.1	17.2	5.55
	163	336.5	14.0	3.1	11.7	4.18
	164	336.5	14.0	3.1	6.0	2.59
	165	336.5	14.0	3.1	8.4	3.58
	166	336.5	14.0	3.1	14.4	4.76
	167	336.5	14.0	3.1	21.1	6.82
21	168	268.6	13.5	6.3	26.0	15.65
	169	268.6	13.5	6.3	21.8	13.95
	170	268.6	13.5	6.3	14.8	9.78
	171	268.6	13.5	6.3	9.7	7.07
	172	268.6	13.5	6.3	5.1	4.53
	173	268.6	13.5	6.3	7.3	5.61
	174	268.6	13.5	6.3	11.7	8.01
	175	268.6	13.5	6.3	16.8	10.65
22	176	268.6	20.4	6.0	22.7	14.20
	177	268.6	20.4	6.0	19.6	12.82
	178	268.6	20.4	6.0	13.4	9.14
	179	268.6	20.4	6.0	9.0	6.66
	180	268.6	20.4	6.0	4.5	4.20
	181	268.6	20.4	6.0	6.5	5.18
	182	268.6	20.4	6.0	10.7	7.60
	183	268.6	20.4	6.0	15.6	10.68
23	184	276.3	20.4	5.8	21.6	12.72
	185	276.3	20.4	5.8	18.7	11.09
	186	276.3	20.4	5.8	13.1	8.22
	187	276.3	20.4	5.8	8.6	6.24

	188	276.3	20.4	5.8	4.3	3.88
	189	276.3	20.4	5.8	6.3	4.69
	190	276.3	20.4	5.8	10.0	6.87
	191	276.3	20.4	5.8	15.2	9.44
24	192	283.2	20.4	5.5	18.4	10.40
	193	283.2	20.4	5.5	17.2	9.76
	194	283.2	20.4	5.5	12.3	7.62
	195	283.2	20.4	5.5	8.1	5.74
	196	283.2	20.4	5.5	3.9	3.56
	197	283.2	20.4	5.5	5.8	4.52
	198	283.2	20.4	5.5	9.1	6.11
	199	283.2	20.4	5.5	14.0	8.65
25	200	304	20.4	5.2	17.8	8.31
	201	304	20.4	5.2	16.7	8.02
	202	304	20.4	5.2	11.5	6.02
	203	304	20.4	5.2	7.6	4.49
	204	304	20.4	5.2	3.7	2.84
	205	304	20.4	5.2	5.5	3.50
	206	304	20.4	5.2	8.8	4.64
	207	304	20.4	5.2	13.1	6.27
26	208	313.3	20.4	4.6	16.9	6.96
	209	313.3	20.4	4.6	15.3	6.56
	210	313.3	20.4	4.6	11.2	5.09
	211	313.3	20.4	4.6	7.6	3.80
	212	313.3	20.4	4.6	3.7	2.54
	213	313.3	20.4	4.6	5.6	3.09
	214	313.3	20.4	4.6	8.6	4.10
	215	313.3	20.4	4.6	12.9	5.70
27	216	324	20.4	4.0	16.5	6.67
	217	324	20.4	4.0	15.2	5.95
	218	324	20.4	4.0	10.9	4.80
	219	324	20.4	4.0	7.2	3.59
	220	324	20.4	4.0	3.5	2.30
	221	324	20.4	4.0	5.2	2.77
	222	324	20.4	4.0	8.3	3.70
	223	324	20.4	4.0	12.4	4.93
28	224	335.5	20.4	4.5	18.8	6.25
	225	335.5	20.4	4.5	15.1	5.46

226	335.5	20.4	4.5	10.7	4.15
227	335.5	20.4	4.5	7.2	3.03
228	335.5	20.4	4.5	3.6	2.17
229	335.5	20.4	4.5	5.2	2.76
230	335.5	20.4	4.5	8.2	3.50
231	335.5	20.4	4.5	12.1	4.82

[a] in unit of molecule cm^{-3}.

[b] The $[CH_2OO]_0$ can be determined by fitting the CH_2OO time traces measured in the experiments in the absence of HCHO with the kinetic model.[10]

[c] The mixing ratio of the gaseous HCHO in the bath gas O_2/N_2 before injection into the reactor was determined using UV absorption spectra and the absorption cross section of HCHO in region 350–358 nm.[11] The $[HCHO]_0$ in the reactor was estimated by the ratio of its flow rate to the total flow rate and the total pressure. Considering the errors of spectral fitting (3 %), UV absorption cross section of HCHO at 350–358 nm (2 %), the flow rates (2 %), temperature (1 %), and pressure (1 %), an overall uncertainty of $[HCHO]_0$ was estimated the to be 4.4 %.

[d] The k_{obs} obtained by fitting of CH_2OO traces with single exponential function.

Supplementary Table 2. Summary of experimental and theoretical results of the rate coefficient for the reactions of CH_2OO with HCHO, CH_3CHO, and CH_3COCH_3.

Reaction	T / K	Experimental rate coefficient [a]	Theoretical rate coefficient [a]	References
CH_2OO + HCHO	296	$(4.11\pm0.25)\times10^{-12}$		This work
CH_2OO + HCHO	268.6–336.5	$(1.91\pm0.15)\times10^{-13}\times$ exp[(1.81 ± 0.04) kcal mol^{-1}/RT]		This work
CH_2OO + HCHO	298		8.3×10^{-13}	Jalan *et al.*[2]
CH_2OO + HCHO	260–350		$4.5\times10^{-13}\times$exp[0.36 kcal mol^{-1}/RT]	Jalan *et al.*[2]
CH_2OO + HCHO	298		6.2×10^{-11}	Long *et al.*[1]
CH_2OO + HCHO	280–350		$2.7\times10^{-14}\times$exp[4.58 kcal mol^{-1}/RT]	Long *et al.*[1]
CH_2OO + CH_3CHO	293	$(9.5\pm0.7)\times10^{-13}$		Taatjes *et al.*[12]
CH_2OO + CH_3CHO	298	$(1.2\pm0.2)\times10^{-12}$		Elsamra *et al.*[13]
CH_2OO + CH_3CHO	298–494	$(3\pm0.8)\times10^{-14}\times$ exp[(2.18 ± 0.65) kcal mol^{-1}/RT]		Elsamra *et al.*[13]
CH_2OO + CH_3CHO	298		5.5×10^{-13}	Jalan *et al.*[2]
CH_2OO + CH_3COCH_3	293	$(2.3\pm0.3)\times10^{-13}$		Taatjes *et al.*[12]
CH_2OO + CH_3COCH_3	298	$(3.0\pm0.6)\times10^{-13}$		Elsamra *et al.*[13]
CH_2OO + CH_3COCH_3	298–494	$(7\pm2.5)\times10^{-15}\times$ exp[(2.22 ± 0.69) kcal mol^{-1}/RT]		Elsamra *et al.*[13]
CH_2OO + CH_3COCH_3	298		3.8×10^{-13}	Jalan *et al.*[2]

[a] in cm^3 molecule^{-1} s^{-1}.

Supplementary Table 3. Kinetic model and rate coefficients employed for product analysis.

	Reaction	Rate coefficient [a]	Ref.
k_{1a} [b]	$CH_2OO + HCHO \rightarrow HCOOH$ ($v = 0$) + HCHO	$y_{HCOOH} \times \alpha \times k_{CH2OO+HCHO}$	*d*
$k_{1a'}$ [b]	$CH_2OO + HCHO \rightarrow HCOOH^{\#} + HCHO$	$y_{HCOOH} \times (1-\alpha) \times k_{CH2OO+HCHO}$	*d*
k_{1b} [b]	$CH_2OO + HCHO \rightarrow CO$ ($v = 0$) + H_2O + HCHO	$y_{CO} \times \beta \times k_{CH2OO+HCHO}$	*d*
$k_{1b'}$ [b]	$CH_2OO + HCHO \rightarrow CO^{\#}$ ($v > 0$) + H_2O + HCHO	$y_{CO} \times (1-\beta) \times k_{CH2OO+HCHO}$	*d*
k_{1c} [b]	$CH_2OO + HCHO \rightarrow$ other products	$(1-y_{HCOOH}-y_{CO}) \times k_{CH2OO+HCHO}$	*d*
k_2	$CH_2OO + CH_2OO \rightarrow 2HCHO + O_2$	8.0×10^{-11}	*10*
k_3	$CH_2OO + I \xrightarrow{+M}$ product	$k_3 = \{4.4\times10^{-29}[M]\times6.7\times10^{-11}\} / \{4.4\times10^{-29}[M]+6.7\times10^{-11}\}$	*10*
k_4	$CH_2OO + HCOOH$ ($v = 0$) $\rightarrow$ products	$1.5\times10^{-11} \times \exp(4.9\ kJ\ mol^{-1}/RT)$	*14*
k_5	$CH_2OO + HCOOH^{\#} \rightarrow$ products	$1.5\times10^{-11} \times \exp(4.9\ kJ\ mol^{-1}/RT)$	*14*
k_6	$HCOOH^{\#} \rightarrow HCOOH$ ($v = 0$)	k_6 fitted [c]	*d*
k_7	$CO^{\#}$ ($v > 0$) $\rightarrow CO$ ($v = 0$)	k_7 fitted [c]	*d*

[a] Rate coefficient in $cm^3\ molecule^{-1}\ s^{-1}$, unless specified, [M] in molecule cm^{-3}.

[b] $k_{1a} + k_{1a'} + k_{1b} + k_{1b'} + k_{1c} = k_{CH2OO+HCHO}$.

[c] Rate coefficient in s^{-1}.

[d] The values obtained in this work.

Supplementary Table 4. Summary of experimental conditions, fitting parameters, and branching yields of HCOOH and CO.

Expt.[a]	Temp. / K	P_T / Torr	$[CH_2OO]_0$ / 10^{13} [b, c]	$[HCHO]_0$ / 10^{15} [b]	y_{HCOOH} [d]	y_{CO} [d]	α	β	k_6 / s^{-1}	k_7 / s^{-1}
1	296	15.1	3.71	4.0	0.43	0.57	0.60	0.45	130	300
2	296	28.3	3.56	3.6	0.47	0.53	0.60	0.50	130	300
3	296	46.1	3.73	3.4	0.50	0.50	0.60	0.50	130	300
4	296	59.7	3.72	3.7	0.52	0.45	0.60	0.50	130	300
5	283	14.7	3.85	2.9	0.37	0.63	0.60	0.45	125	280
6	283	27.4	3.70	2.6	0.43	0.57	0.60	0.45	125	280
7	283	44.1	3.83	2.4	0.52	0.48	0.60	0.45	125	280
8	283	59.4	3.82	2.9	0.54	0.42	0.60	0.45	125	280
9	313	14.5	3.26	2.6	0.40	0.60	0.60	0.50	150	400
10	313	27.0	3.21	2.4	0.43	0.57	0.60	0.50	150	400
11	313	43.3	3.32	2.2	0.46	0.54	0.60	0.50	150	400
12	313	59.1	3.36	2.6	0.47	0.53	0.60	0.50	150	400

[a] Each experiment set was also performed by replacing HCHO to SO_2. The $[SO_2]_0$ of $\sim 4\times10^{14}$ molecule cm^{-3} was used in the experiments. In addition, the CO formed from the photodissociation of HCHO by 248-nm photolysis beam was also determined in each experiment set.

[b] in unit of molecule cm^{-3}.

[c] The concentration of CH_2OO includes $[CH_2OO\ (v = 0)]$ and $[CH_2OO\ (v > 0)]$. In the $CH_2I + O_2$ reaction system, the initial concentration of the I atom of $\sim 8\times10^{13}$ molecule cm^{-3} was estimated in the reaction system.

[d] The y_{HCOOH} and y_{CO} represent the branching ratios for the HCOOH + HCHO and CO + H_2O + HCHO product channels, respectively, in the CH_2OO + HCHO reaction.

Supplementary References

(1) Long, B., Wang, Y., Xia, Y., He, X., Bao, J. L. & Truhlar, D. G. Atmospheric Kinetics: Bimolecular Reactions of Carbonyl Oxide by a Triple-Level Strategy. *J. Am. Chem. Soc.* **143**, 8402–8413 (2021).

(2) Jalan, A., Allen, J. W. & Green, W. H., Chemically activated formation of organic acids in reactions of the Criegee intermediate with aldehydes and ketones. *Phys. Chem. Chem. Phys.* **15**, 16841–16852 (2013).

(3) Elakiya, C., Shankar, R., Vijayakumar S. & Kolandaivel, P. A theoretical study on the reaction mechanism and kinetics of allyl alcohol (CH_2=$CHCH_2OH$) with ozone (O_3) in the atmosphere. *Molecular Physics* **115**, 895–911 (2017).

(4) *Hitran Database* https://hitran.org/ (accessed 2022-11-09).

(5) Schuyler, T. J.; Gohari, S. M. I.; Pundsack, G.; Berchoff, D.; Guzman, M. I. Using a Balloon-Launched Unmanned Glider to Validate Real-Time WRF Modeling. *Sensors* **2019**, *19*, 1914.

(6) Buck Research Manual (1996), updated equation from Buck, A. L. New equations for computing vapor pressure and enhancement factor. *J. Appl. Meteorol.* **1981**, *20*, 1527−1532.

(7) Ruscic, B. Active Thermochemical Tables: Water and Water Dimer. *J. Phys. Chem. A* **2013**, *117*, 11940– 11953.

(8) Lin, L.; Chang, H.; Chang, C.; Chao, W.; Smith, M. C.; Chang, C.; Lin, J. J.; Takahashi, K. Competition between H_2O and $(H_2O)_2$ reactions with CH_2OO/CH_3CHOO. *Phys. Chem. Chem. Phys.* **2016**, *18*, 4557−4568.

(9) Bernath, P.; Steffen, J.; Crouse, J.; Boone, C. *Atmospheric Chemistry Experiment SciSat Level 2 Processed Data, v4.0. Federated Research Data Repository.* https://doi.org/10.20383/101.0291 (accessed 2022-11-09).

(10) Mir, Z. S.; Lewis, T. R.; Onel, L.; Blitz, M. A.; Seakins, P. W.; Stone, D. CH_2OO Criegee intermediate UV absorption cross-sections and kinetics of CH_2OO + CH_2OO and CH_2OO + I as a function of pressure. *Phys. Chem. Chem. Phys.* **2020,** *22,* 9448–9459.

(11) Meller, R.; Moortgat, G. K. Temperature dependence of the absorption cross sections of formaldehyde between 223 and 323 K in the wavelength range 225-375 nm. *J. Geophys. Res. D* **2000**, *105*, 7089–7101.

(12) Taatjes, C. A., Welz, O., Eskola, A. J., Savee, J. D., Osborn, D. L., Lee, E. P. F., Dyke, J. M. D., Mok, W. K., Shallcross D. E. & Percival, C. J. Direct measurement of Criegee intermediate (CH_2OO) reactions with acetone, acetaldehyde, and hexafluoroacetone, *Phys. Chem. Chem. Phys.* **14**, 10391–10400 (2012).

(13) Elsamra, R. M., Jalan, A., Buras, Z. J., Middaugh, J. E. & Green, W. H. Temperature and pressure-dependent kinetics of CH_2OO + CH_3COCH_3 and CH_2OO + CH_3CHO: Direct measurements and theoretical analysis, *Int. J. Chem. Kinet.* **48**, 474–488 (2016).

(14) Peltola, J.; Seal, P.; Inkilä, A.; Eskola A. Time-resolved, broadband UV-absorption spectrometry measurements of Criegee intermediate kinetics using a new photolytic precursor:

unimolecular decomposition of CH_2OO and its reaction with formic acid. *Phys. Chem. Chem. Phys.* **2020,** *22,* 11797–11808.
